# Retrorectal Bronchogenic Cyst With a Sacrococcygeal Surgical Approach

**DOI:** 10.7759/cureus.31583

**Published:** 2022-11-16

**Authors:** Daniel de Barcellos Azambuja, Bruna Oliveira Trindade, Paulo Valdeci Worm, Fares Hassan Hamaoui, Júlia Iaroseski

**Affiliations:** 1 Colorectal Surgery, Santa Casa de Misericórdia de Porto Alegre, Porto Alegre, BRA; 2 Medical School, Federal University of Health Sciences of Porto Alegre, Porto Alegre, BRA; 3 Neurosurgery, Santa Casa de Misericórdia de Porto Alegre, Porto Alegre, BRA

**Keywords:** case report, surgery, sacrococcygeal, retrorectal, bronchogenic cyst

## Abstract

We present a case of a bronchogenic cyst in the retrorectal space that was successfully treated with a unique sacrococcygeal surgical approach, thus avoiding abdominal access. This report aims to enhance the literature with our technique and to help the scientific community in treating future retrorectal bronchogenic cyst cases. A 19-year-old man presented to the hospital with testicular pain refractory to over-the-counter analgesics. A cyst lesion in the presacral region was found during pelvic magnetic resonance imaging. We identified the cyst’s epithelium as the respiratory type, containing ciliated pseudostratified columnar epithelium with foci of squamous metaplasia. The bronchogenic cyst is abnormal from the primitive foregut, and its location on the retrorectal site is rare. Patients with this type of lesion are usually asymptomatic, and discovery is commonly incidental during imaging exams. Excision is advised, and the prognosis is good after retroperitoneal bronchogenic cyst removal. Our description of the topic is essential to assist future similar cases.

## Introduction

A bronchogenic cyst is a congenital abnormal formation from the primitive foregut [1]. During the third to seventh week of development [2], bronchogenic cysts surge from the tracheobronchial of the primitive foregut as abnormal budding [3]. This formation can continue attached to the primitive foregut, promoting a location associated with the tracheobronchial tree or esophagus [3]. They are typically located in the posterior mediastinum and rarely detected in the retroperitoneum [4].

Before the fusion of pleuroperitoneal membranes, a part of the tracheobronchial tree can be separated entirely and migrate, giving rise to a retroperitoneal bronchogenic cyst [2]. However, retroperitoneal bronchogenic cysts are usually situated on the superior border of the pancreas or near the left adrenal gland [1].

Overall, the presence of tumors in the retrorectal space is rare [5]. Most of them are benign, and the malignant ones are more common in children than in adults [5]. This site has embryologic remnants from various tissues and can be the location of lesions with multiple origins [5,6]. The congenital cyst type is more frequent [6] and is less likely to be malignant than solid lesions [5]. The diagnosis is commonly performed incidentally during imaging exams [1]. Excision is advised to establish a diagnosis, treat any symptoms, and avoid complications, even when infected [3]. The prognosis after retroperitoneal bronchogenic cyst removal is good, with no reported recurrence [7].

The current study presents the case of a bronchogenic cyst in the retrorectal space of a 19-year-old man, which was successfully resected by a sacrococcygeal surgical approach.

## Case presentation

**Figure 1:**
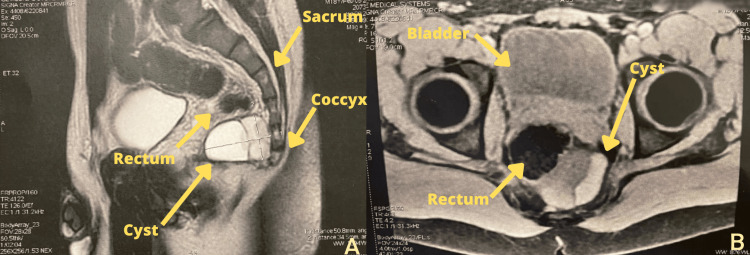
Cyst identification on magnetic resonance imaging (MRI) (A) Sagittal cross-section view; (B) axial cross-section view

A 19-year-old man presented to the hospital with testicular pain refractory to over-the-counter analgesics. The patient denied any past medical condition or any congenital affections. The patient denied previous imaging investigation or surgical intervention of the abdomen or pelvis. The physical exam including digital rectal examination, genital and testicular evaluation, and abdominal palpation did not show any important findings that would lead to a diagnosis. The family medical history investigation was not indicative of any rare syndrome or specific neoplasia. Due to the chronic character of pain and a very slow progression of symptoms, we discarded the following conditions from the differential diagnosis: testicular torsion, epididymo-orchitis, or orchitis. The genital physical exam was not indicative of a testicular tumor. Urinalysis and complete blood count were also normal. The patient was admitted to the surgical ward for further workup. Pelvic magnetic resonance imaging (MRI) revealed an incidental finding of a homogenous retrorectal cyst measuring 5x4x4 cm in front of the sacrum without any signs of invasion into the surrounding structures (Figure 1). The differentials for the cyst were benign cyst, meningocele, meningomyelocele, and bronchogenic cyst.

**Figure 2:**
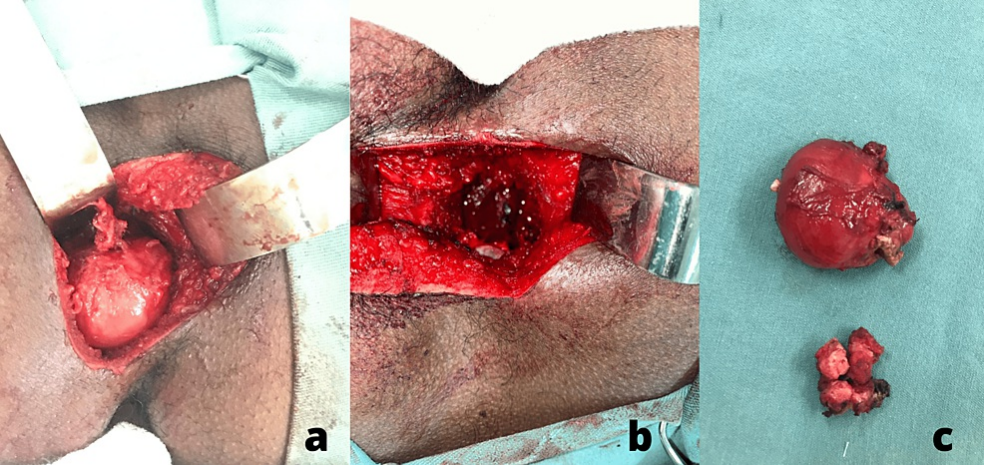
Resection with sacrococcygeal surgical approach (a) Exposure of the cyst, (b) resection site after cyst removal, (c) surgical piece

The neurosurgical and proctology team recommended surgical resection. After written informed consent was obtained from the patient, an open surgical excision was performed with an intergluteal cleft incision. After coccygectomy, we found the cystic lesion adhered to the deep planes and close to the colon with dense intracystic content (Figure 2A). Following the surgical excision of the cyst (Figure 2B), we sutured the colon to the pelvic side wall (colorrhaphy) and repaired the sphincter with a muscle flap from the levator ani. After hemostasis, we closed the wound in reverse order and applied antiseptic dressing. The patient recovered with no complications in the postoperative period, and with complete preservation of sphincter functionality.

**Figure 3:**
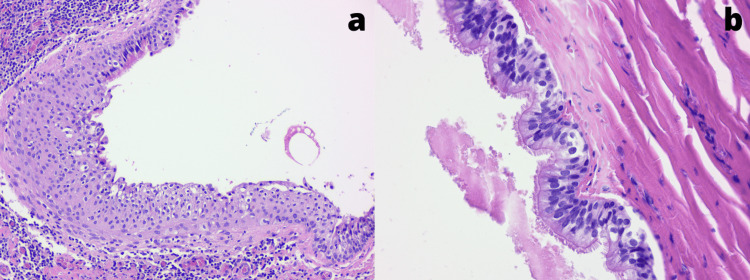
Histology of the bronchogenic cyst (a) Overview of the microscopic view: the cyst's epithelium wall presenting squamous metaplasia, suggesting a respiratory origin, (b) detail of the microscopic view: ciliated pseudostratified columnar epithelium found in the cyst's wall

From the surgical piece (Figure 2C), histopathology examination revealed a cyst with dense chronic inflammatory lesions lined by ciliated pseudostratified columnar epithelium (respiratory) with foci of squamous metaplasia (Figure 3). This confirmed the diagnosis of a benign bronchogenic cyst with negative margins.

After three weeks, the patient remained asymptomatic and an MRI was performed, which confirmed no cyst recurrence. Follow-up visits continue every eight months to evaluate reoccurrence and symptomatology, which have both been negative to date.

## Discussion

Patients with bronchogenic cysts are usually asymptomatic [8] but can present symptoms such as pain and constipation [9]. Typically, the lesion has a diameter of fewer than 5 cm [8], with a correlation between age, growth, and pain [10]. In the retroperitoneal space, this specific lesion location is usually found in the corpus of the pancreas and left adrenal gland region [11]. Our patient was a 19-year-old man who came to the hospital because of testicular pain and incidentally discovered a cyst, sized approximately 5 cm.

Commonly, the use of radiological modality facilitates preoperative differential diagnosis [12]. Abdominal CT scans and ultrasounds can determine an abdominal mass's size and location, as well as its solid and cystic properties [12]. However, MRI offers more accurate imaging than CT [13]. Due to the abnormal clinical presentation of the retroperitoneal bronchogenic cysts, they are often incidentally detected through CT scans or MRIs [8]. In the present case, an pelvic MRI revealed an incidental homogenous retrorectal cyst that further histopathology confirmed as a bronchogenic cyst.

Most bronchogenic cysts are lined at least partially by ciliated cuboidal to the pseudostratified columnar epithelium and are usually filled with mucus [2]. Besides that, some bronchial components can be found in the cyst's wall, such as cartilage, smooth muscle, elastic fibers, fibrous tissue, and seromucinous glands [2]. In our case, we found compatible histology described in the literature, a ciliated pseudostratified columnar epithelium. In addition, the cyst in this report has foci of squamous metaplasia, a standard reactive change of the bronchial epithelium that is not considered preneoplastic [14]. Malignancy is rare in retroperitoneal bronchogenic cysts [2].

Despite its benign nature, surgery is the treatment for bronchogenic cysts [3]. Early cyst removal is encouraged because previously infected cysts are riskier and more difficult to remove [3]. In addition, excision establishes a diagnosis, treats any symptoms, and avoids complications [3]. According to the literature, surgeons can use several resection techniques depending on the cyst location [15]. Among them are the anterior surgical approach, posterior surgical approach, and combination abdominoperineal approach [16]. Generally, a posterior approach is favored for lesions below the level of S3, while an anterior or mixed approach is preferred for those above the level of S3 [16]. Our surgical team chose a sacrococcygeal approach, following literature recommendations. 

After resectioning the retroperitoneal bronchogenic cyst, the prognosis is favorable, and there have been no documented recurrences [7]. Even though there has been no recorded recurrence, we scheduled follow-up visits for our patient to evaluate reoccurring and symptomatology. CT scans are appropriate for evaluating retroperitoneal disease because they offer distinct sectional pictures of the organs and retroperitoneal compartments [17]. There is no consensus about how long we should follow the patient. Nonetheless, Yuan et al. [4] showed in a literature review that some studies follow their patient for up to four years.

Our case stands out because of the singularity of the lesion, a retrorectal bronchogenic cyst. Until this moment there is not any data about the exact incidence or prevalence of sacral location of bronchogenic cyst, demonstrating its rarity. However, two similar cases can be found in the previous literature, confirming the uniqueness of the present case. Ko et al. reported a similar sacral location of a congenital bronchogenic cyst, but due to its intraspinal site, the lesion also had subarachnoid space invasion [18]. Pasquer et al. also described a sacral coccyx bronchogenic cyst diagnosis in a patient initially treated for pilonidal cyst presenting recurrence [15]. The authors recommended a double surgical approach using sacrococcygeal and abdominal access.

## Conclusions

Our study reports a case of a rare presentation of a retrorectal bronchogenic cyst, which we removed by a sacrococcygeal surgical approach to avoid abdominal access and a more invasive procedure. The uniqueness of this case is because of the particular clinical presentation of a bronchogenic cyst; therefore, the description of the topic is necessary to enhance the literature with our technique and to help the scientific community in future similar cases.
